# Three new clades of putative viral RNA-dependent RNA polymerases with rare or unique catalytic triads discovered in libraries of ORFans from powdery mildews and the yeast of oenological interest *Starmerella bacillari*s

**DOI:** 10.1093/ve/veac038

**Published:** 2022-04-23

**Authors:** Marco Forgia, M Chiapello, Stefania Daghino, D Pacifico, D Crucitti, D Oliva, M Ayllon, M Turina, M Turina

**Affiliations:** Institute for Sustainable Plant Protection (IPSP), CNR, Strada delle Cacce 73, Torino 10135, Italy; Institute for Sustainable Plant Protection (IPSP), CNR, Strada delle Cacce 73, Torino 10135, Italy; Institute for Sustainable Plant Protection (IPSP), CNR, Strada delle Cacce 73, Torino 10135, Italy; Institute of Biosciences and Bioresources (IBBR), CNR, Corso Calatafimi 414, Palermo 90129, Italy; Institute of Biosciences and Bioresources (IBBR), CNR, Corso Calatafimi 414, Palermo 90129, Italy; Dipartimento di Scienze Agrarie, Alimentari e Forestali (SAAF), Università degli Studi di Palermo, Viale delle Scienze, Palermo 90128, Italy; Istituto Regionale del Vino e dell’Olio (IRVO), Via Libertà 66, Palermo 90143, Italy; Centro de Biotecnología y Genómica de Plantas, Universidad Politécnica de Madrid (UPM)-Instituto Nacional de Investigación y Tecnología Agraria y Alimentaria (INIA), Campus de Montegancedo, Pozuelo de Alarcón, Madrid 28223, Spain; Departamento de Biotecnología-Biología Vegetal, Escuela Técnica Superior de Ingeniería Agronómica, Alimentaria y de Biosistemas, Universidad Politécnica de Madrid (UPM), Campus Ciudad Universitaria Av. Puerta de Hierro, nº 2 - 4, Madrid 28040, Spain; Institute for Sustainable Plant Protection (IPSP), CNR, Strada delle Cacce 73, Torino 10135, Italy; Institute for Sustainable Plant Protection (IPSP), CNR, Strada delle Cacce 73, Torino 10135, Italy

**Keywords:** viral RdRP, evolution, mycovirus, ORFan, ormycovirus

## Abstract

High throughput sequencing allowed the discovery of many new viruses and viral organizations increasing our comprehension of virus origin and evolution. Most RNA viruses are currently characterized through similarity searches of annotated virus databases. This approach limits the possibility to detect completely new virus-encoded proteins with no detectable similarities to existing ones, i.e. ORFan proteins. A strong indication of the ORFan viral origin in a metatranscriptome is the lack of DNA corresponding to an assembled RNA sequence in the biological sample. Furthermore, sequence homology among ORFans and evidence of co-occurrence of these ORFans in specific host individuals provides further indication of a viral origin. Here, we use this theoretical framework to report the finding of three conserved clades of protein-coding RNA segments without a corresponding DNA in fungi. Protein sequence and structural alignment suggest these proteins are distantly related to viral RNA-dependent RNA polymerases (RdRP). In these new putative viral RdRP clades, no GDD catalytic triad is present, but the most common putative catalytic triad is NDD and a clade with GDQ, a triad previously unreported at that site. SDD, HDD, and ADD are also represented. For most members of these three clades, we were able to associate a second genomic segment, coding for a protein of unknown function. We provisionally named this new group of viruses ormycovirus. Interestingly, all the members of one of these sub-clades (gammaormycovirus) accumulate more minus sense RNA than plus sense RNA during infection.

## 1. Introduction

Recent advancements in characterizing virus sequences from complex samples directly by high throughput sequencing (HTS) of DNA or RNA has greatly increased our knowledge of virus diversity: a number of investigations have characterized viruses in the ocean and soil (Brum et al. 2015; Gregory et al. 2019; Starr et al. 2019; Vlok, Lang, and Suttle 2019), viruses infecting invertebrate hosts (Li et al. 2015; Shi et al. 2016; Kaefer et al. 2019), and plants (Dolja, Krupovic, and Koonin 2020); furthermore recent targeted efforts have also increased the knowledge of RNA viruses in prokaryotes (Callanan et al. 2021). Finally, two distinct efforts to mine public databases (particularly metatranscriptomes) for RNA viruses resulted in order of magnitude increases in viral taxa corresponding to the genus/species level, with much-expanded knowledge of virus diversity (Edgar et al. 2022; Neri et al. 2022).

Fungi and oomycetes, in particular the obligatory biotrophic ones, have been still minimally investigated for their overall viral diversity. Nonetheless, a number of recent studies have shown an unsuspected diversity of viral genomic organizations (ssDNA, +ssRNA, -ssRNA, and dsRNA) and lifestyles also in fungi (Yu et al. 2010; Kanhayuwa et al. 2015; Marzano and Domier 2016; Deakin et al. 2017; Hisano et al. 2018; Sato et al. 2020; Sutela et al. 2020; Chiapello et al. 2020a; Ruiz-Padilla et al. 2021).

Altogether, these findings brought to a new hypothesis about virus origin and evolution, suggesting that viruses probably originate from primordial replicators, recruiting capsids from hosts (Krupovic, Dolja, and Koonin 2019).

Given the polyphyletic nature of viruses and the lack of a common marker gene for metabarcoding current searches of new viruses rely on similarity-based searches of existing virus databases mostly through Basic Local Alignment Search Tool (BLAST) algorithms (Altschul et al. 1990).

This approach has some inherent limitations due to the fact that most viruses code for proteins that are not conserved enough to be detected through similarity searches using BLASTX (Gish and States 1993) or other profile-based methods such as HMMER (Finn et al. 2015). A further evolution that can improve the detection of distantly related proteins is based on structural alignment algorithms such as Phyre2 (Kelley et al. 2015), but this approach has the downside of a high computational requirement that makes it unsuitable for HTS applications.

The viral dark matter (Webster et al. 2015) can also be investigated with other targeted approaches such as those that previously brought to the discovery of the Quenyaviruses in *Drosophila* (Obbard et al. 2020), which relied also on sRNA libraries, enriched in virus-derived sRNA from the host antiviral immunity.

Previously, in order to find possible biocontrol agents in the virome associated to biotrophic fungi/oomycetes and vector insects, we developed a pipeline to look at ORFan coding segments that are mapped by both +RNA and -RNA sequencing reads, which is an indication of the possible presence of both +RNA and -RNA transcripts, a hallmark of RNA virus infection. We have recently applied this pipeline in a number of virome characterization projects and we discovered virus-encoded ORFan segments or genomes that were later confirmed to be virus sequences: among our most relevant findings is the discovery of nucleocapsid-encoding fragments associated to mycobunya-like viruses that allowed to complete the genome sequence of these viruses now included in the family *Discoviridae* (https://talk.ictvonline.org/, last accessed on 14 April 2022); the discovery of a second segment associated to a yue-like and orfanplasmo-like RNA-dependent RNA polymerase (RdRP) from thrips viromes (Chiapello et al. 2021); the discovery of the narna-like clade called ‘orfanplasmovirus’ form fungi/oomycetes (Chiapello et al. 2020a), and the discovery of the ambivirus clade (Sutela et al. 2020), a group of ambisense viruses with unprecedented genome organization that could require an expansion of the Baltimore’s classification (Koonin, Krupovic, and Agol 2021).

The scope of this work is the analysis of some ORFan segments, found in a new dataset of total RNA obtained from powdery mildew lesions from vegetable crops and grapevine. We will provide evidence that we characterized three new clades of viral RdRPs with clear structural conservation with previously characterized viral RdRPs, but with very limited protein sequence conservation, which prevented their detection through similarity searches and prevents their reliable placement in current versions of the monophyletic RNA virus phylogenetic tree. We propose for these new related virus clades, the name ‘ormycovirus’ (ORFan mycovirus). Through homology search, we detected other examples of the new viruses in fungal-related transcriptome shotgun assembly (TSA) from the National Centre for Biotechnology Information (NCBI) and in a collection of isolates from the yeast of oenological interest *Starmerella bacillaris* (synonym *Candida zemplinina*), which we have recently characterized (Crucitti et al. 2022). Further analysis of these new clades allowed us to provide evidence of a second RNA segment associated to the ormycovirus RdRP-encoding fragment, coding for a conserved protein of unknown functions.

## 2. Methods

### 2.1 Original samples, sequencing, and trinity libraries

Samples corresponding to powdery mildew lesions of vegetable crops and of grapevines were collected in 2018 (fungal species displayed in sample metadata Supplementary Table S1). In particular, the libraries obtained from the downy mildew of grapevine are the same as those described in our previous publication (Chiapello et al. 2020a). Sample preparation and total RNA extraction from each sample were carried out as previously detailed (Chiapello et al. 2020a); samples were mixed in pools PM-A and PMG-A corresponding to vegetable and grapevine powdery mildew lesions, respectively, and were sent to Macrogen Inc. (Seoul, Republic of Korea) for Illumina TrueSeq Stranded library preparation and sequencing. The obtained 150-bp paired-end reads were deposited in the Sequencing Read Archive (SRA) with the following accession numbers: PM-A (BIOSAMPLE SAMN21876559) and PMG-A (BIOPROJECT PRJNA803385).

Reads were cleaned from the library adaptor, artefacts, and low-quality base call values and assembled by Trinity software version 2.9.1 (Grabherr et al. 2011) following a pipeline described in detail previously (Chiapello et al. 2020a). Libraries from Chiapello and coworkers study on the mycoviruses associated to *Plasmopara viticola* lesions are available on NCBI SRA (BiOSAMPLE from SAMN14402112 to SAMN14402118, libraries DMG-A to DMG-G).

In this work, we also screened for specific virus presence a collection of *S. bacillaris* yeast (formerly known as *C. zemplinina*) provided by the Istituto Regionale del Vino e dell’Olio (IRVO; Supplementary Table S2) and grown following conditions previously described (Crucitti et al. 2022). *S. bacillaris* was grown on Yeast Extract-Peptone-Dextrose (YPD) solid medium or YPD liquid medium (10 g/l of Yeast extract, 20 g/l of Peptone and 20 g/l of Glucose, the latter added after autoclaving from a filtered stock solution; solid media was added with 20 g/l of Agar) for RNA extraction and particle purification. The collection was maintained using glycerol stock at −80°C, produced by adding 300 μl of 50 per cent glycerol to 700 μl of overnight yeast culture on liquid YPD.

### 2.2 ORFan searches and homology among ORFans

The schematic representation of the pipeline used to detect and characterize viral ORFan is shown in Supplementary Fig. S1. Briefly, a similarity search was performed with DIAMOND software (v2.0.8) (Buchfink, Xie, and Huson 2015) using as queries, the assembled contigs from each library and as subject, the NCBI non-redundant database (April 2021). The resulting hits were discarded while the contigs with no matches were kept and selected based on nucleic acid size, with only the sequences longer than 1,000 bp passing this filter step (being 1,000 bp, a common threshold for genomic segments of mycoviruses). Among these selected sequences, only the ones that encode proteins of more than 15 kDa were kept (the largest conserved protein encoded by a segment > 1,000 bp is rarely below this molecular mass threshold). These sequences constituted the first draft pool of ORFans. Since a TrueSeq stranded method was used for cDNA synthesis, the information about the read orientation was available, so the reads were mapped on ORFan sequences with bowtie 2 and Samtools (Li et al. 2009; Langmead and Salzberg 2012) taking into account their orientation, i.e. whether they mapped in sense or antisense orientation and displayed graphically with Tablet software (Milne et al. 2013). A typical feature of replicating viruses is the presence of a minus and plus sense genomic template for replication. Accordingly, all the contigs that showed only positive or only negative reads were discarded. The final pool of ORFan sequences was created and it was further analysed for protein similarity among themselves with MAFFT (v7.475).

### 2.3 Mining available fungal transcriptomic and metatranscriptomic databases

In order to identify other homologous ormycovirus from NCBI transcriptomic databases, all 172 available fungal Transcriptome Shotgun Assemblies (TSA) have been downloaded (May 2021). Ormycovirus-like sequences were searched in each TSA using DIAMOND software.

We also searched for ormycovirus presence in the RNA virus databases resulting from a very large metatrascriptomes (RVMT) database that was recently made public (Neri et al. 2022) through DIAMOND software. The sequences showing a hit against ormycovirus RdRP were retrieved and used to perform a phylogenetic analysis (only those with complete coding palm domains). Information on the sample of origin for each of the identified ormycovirus in the RVMT database is shown in Supplementary Table S3.

### 2.4 Protein alignments, phylogenetic analysis, and identity matrixes

Ormycoviruses related RdRPs were aligned using MAFFT (Katoh, Rozewicki, and Yamada 2019) (online version—default parameters) at the EBI Web Services (Madeira et al. 2019). Aligned sequences were subsequently analysed with IQ-TREE software (Trifinopoulos et al. 2016) to produce a phylogenetic tree (maximum likelihood, automatic model selection—default parameters). The latter was visualized with MEGA (v11) (Kumar et al. 2018). Identity matrixes were elaborated with Discovery studio visualizer using as input MAFFT alignments. Identity matrix pictures were produced with reshape2 (Wickham 2007) and tidyverse (Wickham et al. 2019) libraries on R software (R Development Core Team, RFFSC 2011).

### 2.5 In silico structural prediction

In silico structural prediction was performed using a recently developed automatic AlphaFold2 method (Jumper et al. 2021). AlphaFold analysis was carried out in ColabFold (Mirdita, Ovchinnikov, and Steinegger 2021), a Jupyter Notebook (Kluyver et al. 2016) on Google Drive using a slightly simplified version of AlphaFold2. Protein structure figures were created using PyMOL (v2.5.2) (DeLano 2002). Swiss-model (Waterhouse et al. 2018) was used as a second method to confirm the results obtained with AlphaFold using default options on the web browser.

### 2.6 RACE analysis to determine viral RNA termini

Determination of the 5ʹ and 3ʹ ends from Erysiphe lesion associated ormycovirus 1 (ElaOMV1) was obtained through the rapid amplification of cDNA ends (RACE) analysis. The protocol described in detail in literature (Rastgou et al. 2009) consists of the synthesis of the cDNA from infected RNA samples using specific primers (Supplementary Table S4) and SuperScript IV reverse transcriptase (Thermo Fisher, MA, USA). RNA was then removed using RNAse H (Thermo Fisher, MA, USA), and obtained cDNA was purified using Zymo clean and concentrator kit (Zymo Research, CA, USA), eluting in 10 μl. Resulting cDNA was divided into two reactions dATP and dGTP that were used to add a polyA or polyG tail using terminal deoxynucleotidyl transferase (Promega, WI, USA). cDNA was again purified using Zymo clean and concentrator kit in 25 μl, and 1 μl was used in PCR to amplify ElaOMV1 ends with a polyT or polyC primer and a specific primer (Supplementary Table S4). Resulting PCR bands were isolated from gel, purified with Zymo gel DNA recovery kit (Zymo Research, CA, USA), ligated in the pGEMT vector (Promega, WI, USA) and cloned in DH5α chemical competent cells. Positive clones were sequenced by Biofab s.r.l. (Rome, Italy).

For the RNA segment corresponding to Starmerella bacillaris ormycovirus 1 (SbOMV1), a different RACE method was used in order to obtain the full genome sequence. In this case, a modified version of the adaptor ligation mediated RACE protocol presented by Suzuki et al. (Suzuki et al. 2004) was set up to perform RACE reactions starting from the total RNA of the infected host instead of the purified dsRNA. About 2 μg of blocked adaptor (Supplementary Table S4: BlockedAdpt) was ligated to the 3ʹ end of 1 μg of total RNA from *S. bacillaris* Cz12 isolate using T4 RNA ligase (Takara, Japan) as described by the manufacturer’s protocol. Total RNA was cleaned from a reaction buffer using Spectrum Plant Total RNA Kit (Merk, Germany) and cDNA was produced using SuperScript IV reverse transcriptase with a specific primer complementary to the adaptor ligated (Supplementary Table S4: ComplAdapt). Obtained cDNA was diluted 1:5 in deionized water and 1 μl was used for PCR using a specific primer and the primer complementary to the adaptor. Obtained bands were cloned and sequenced as described above.

### 2.7 Molecular analysis of virus segments

The full-length sequence of ElaOMV1 was amplified using specific primers complementary to the terminal sequences and Phusion high fidelity DNA polymerase (NEB, MA, USA). Obtained bands were purified as described for RACE fragments, ligated in pCR blunt vector (Thermo Fisher, MA, USA) and sequenced by the Sanger approach (BioFab srL, Rome, Italy).

Quantitative RT-PCR (qRT-PCR) analyses were performed in two steps: cDNA was synthesized with high-capacity cDNA reverse transcription kit (Thermo Fisher, MA, USA); qPCRs were performed using iTaq universal sybr green supermix on a CFX Connect apparatus (Bio-rad, CA, USA) in 10 μl total volume. All the primers designed to perform qRT-PCR were computed by Primer3 (Untergasser et al. 2012) with amplicon size range parameter between 70 and 120 nucleotides (Supplementary Table S4).

### 2.8 Virus particle purification and electron microscopy analysis

Electron microscope observations were made to detect evidence of viral particle for SbOMV1. *S. bacillaris* isolates Cz12, Cz16, Cz21, and Cz25 were chosen for the presence or absence of SbOMV1, the mitovirus and a totivirus previously described (see Results section below). Isolates were grown in 5 ml of YPD overnight at 30°C and 180 rpm. Yeasts were precipitated at 5,000 g for 5 minutes and pellets were resuspended in 1 ml of water. Resuspended yeasts were transferred in 2-ml tubes, pelleted as before and the pellets were frozen in liquid nitrogen. Silica beads (0.5 mm diameter) were added to each tube and the pellets were broken with a bead beater (MP biochemicals, USA). One ml of 0.25 M phosphate buffer pH 7 was added and cell debris was pelleted with a quick spin in a microcentrifuge. Supernatant was diluted and prepared for TEM observation: each sample was absorbed on carbon and formvar-coated grids and stained with 0.5 per cent Uranyl acetate and observations were made on a Philips CM10 electron microscope (Philips, The Netherlands).

A partial cell fractionation protocol was performed on Cz12 and Cz25 isolates to observe differences in the distribution among fractions compared to the totivirus present in both isolates under investigation. Ten ml of liquid YPD culture was precipitated at 4,000 rpm in a GSA rotor for 1 minute and the pellets were resuspended in 4 ml of deionized water. The precipitation step was repeated, and yeasts were resuspended in 4 ml of 1 M sorbitol. Cultures were again precipitated and resuspended in 4 ml of Sorbitol-Citrate-EDTA-mercaptoethanol (SCEM) buffer (1 M sorbitol, 0.1 M sodium citrate, 10 mM EDTA pH. 8. Buffer is added with 2 μl of 2-mercaptoethanol for each millilitre prior use). OD is measured and 500 U of SCEM resuspended lyticase was added (Merck, Germany). Cultures were kept at 30°C for 1 hour with gentle shaking to produce protoplasts. After incubation, OD was measured again: protoplast production is complete when OD reaches 10 per cent of the initial value. Protoplasts were then pelleted (6,000 rpm for 1 minute at 4°C) and resuspended in 1 ml of 1 M sorbitol; the precipitation step was repeated, and protoplasts were resuspended in lysis buffer (50 mM TrisHCl pH 8, 10 mM EDTA pH 8, 10 mM DTT, 1 per cent TritonX) and vortexed. Fifty  μl were collected from each sample and called sample A. Samples were then centrifuged at 3,000 *g* for 10 minutes at 4°C. Supernatants were collected and centrifuged at 20,000 *g* for 15 minutes, and pellets obtained were collected and called sample P1. Supernatants were centrifuged at 100,000 rpm for 1 hour and 30 minutes in a TL100 ultracentrifuge (Beckman Coulter, CA, USA) and pellets and supernatants were collected and called PU (pellets) and SU (supernatants). Each fraction collected was used for RNA extraction as described above. RNAs were treated with DNAse I (Thermo Fisher, MA, USA) and cDNA were synthesized with High-Capacity cDNA reverse transcription kit (Thermo Fisher, MA, USA).

## 3. Results

### 3.1 Detection of a new clade of ORFAN proteins from different fungi

**Figure 1.**
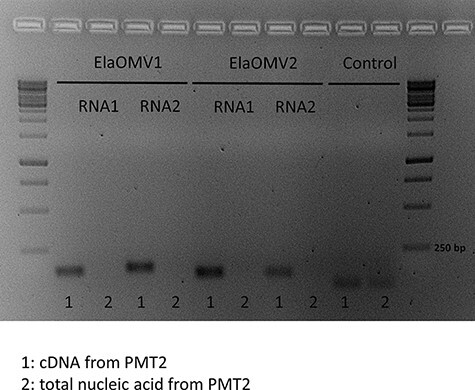
Gel electrophoresis of qRT-PCR fragments obtained from ormycoviruses infecting powdery mildew of tomato. Each genome fragment was amplified with specific PCR primers on cDNA (1) or total nucleic acids without reverse transcription (2) from sample PMT2. Primers detecting *Leveillula taurica* succinate dehydrogenase subunit C were used as control and bands were obtained on both cDNA and total nucleic acids. Primer used is listed in Supplementary Table S4 (ElaOMV1_RNA1_For/Rev, ElaOMV1_RNA2_For/Rev, ElaOMV2_RNA1_For/Rev, ElaOMV2_RNA2_For/Rev, LT_Suc_Real).

Our search of ORFan contigs in the powdery mildew of horticultural plants detected a number of segments that had no match in the databases (NCBInr). Among them, we identified a 3,172 bp contig coding for a single open reading frame (ORF) encoding for a 966 amino acids protein with a calculated molecular weight of 115.74 kDa. Interestingly, this ORFan was a homologue of a recently characterized ORFan from *S. bacillaris* (Crucitti et al. 2022). Given these premises, we investigated the possibility that this segment is part of an RNA viral genome, an indication of such occurrence is the lack of DNA corresponding to the segment in the biological sample. For this purpose, we first detected the contig in specific samples from the pooled NGS library through qRT-PCR finding that the segment is present in one isolate of powdery mildew infecting tomato (PMT2, Supplementary Table S1). We then confirmed the absence of DNA associated to the RNA contig, since the detection through qPCR was only possible in the cDNA from PMT2 and not when total nucleic acid without reverse transcription was used as a template, suggesting a possible viral origin of this segment (Fig. 1). Given its possible viral origin, we named the sequence ElaOMV1 (ormycovirus as the contraction for ‘ORFan mycovirus’). In analogy, the ORFan2 contig previously characterized in *S. bacillaris* isolate Cz12 (Crucitti et al. 2022) is named here SbOMV1.

**Figure 2.**
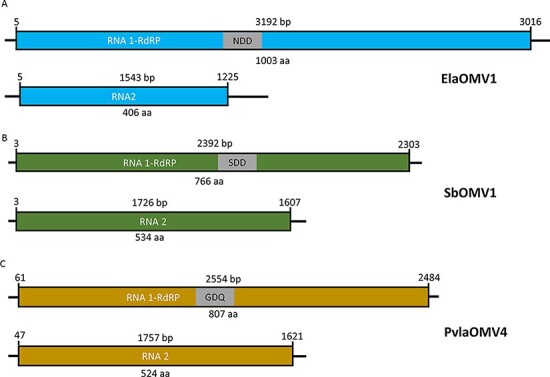
Ormycovirus genome organization. Diagrams show schematic genomes from all the three ormycovirus groups identified: A) genome organization of ElaOMV1 from alphaormycoviruses, B) genome organization of SbOMV1 from betaormycoviruses, C) genome organization of Plasmopara viticola lesion-associated ormycovirus 4 (PvlaOMV4) from gammaormycoviruses. RdRP = RNA-dependent RNA polymerase. For each RNA1 fragment, the palm-domain C motif triplet is shown in the grey box. Numbers at the beginning and at the end of the ORF box show the starting and ending position of the ORF on the genomic fragment.

The RACE analysis was performed to determine the complete sequence of ElaOMV1 on both 5ʹ and 3ʹ ends. From the results obtained, we characterized the full-length sequence of ElaOMV1 that is displayed in Fig. 2. Primers based on RACE results (Supplementary Table S4) were designed to amplify the complete 3,192 bp viral genome from cDNA obtained with the same specific primers to confirm through Sanger sequencing the assembly obtained in silico (Fig. 2). Surprisingly, RT-PCR showed two bands: one at the expected size (around 3 kbp) and a second band at around 1.5 kbp (Supplementary Fig. S2). Since the unspecific 1.5 kbp band could result from a putative additional viral fragment with end sequences conserved with ElaOMV1, we cloned and sequenced it in order to characterize it. This brought us to identify a second ORFan contig called ORFan2, a 1,543 bp long sequence encoding for a putative protein of 406 amino acids (Fig. 2), which supports the hypothesis of a bisegmented nature of ElaOMV1 genome. The RACE analysis on this fragment indeed confirmed that RNA1 and RNA2 had conserved ends, with only one difference in the first twenty nucleotides (Supplementary Fig. S3).

**Figure 3.**
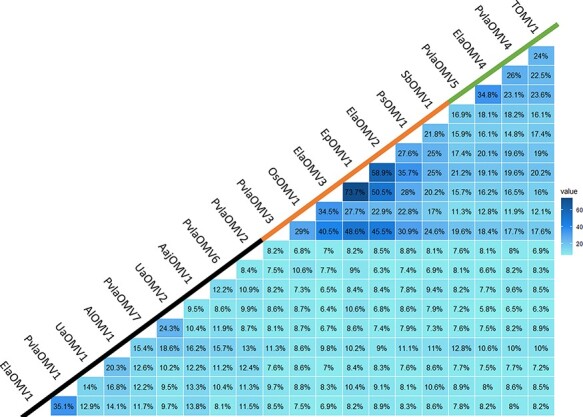
Pairwise identity matrix obtained from putative proteins encoded by ormycovirus RNA1. Protein alignment was built using MAFFT and identity is shown in percentage for each comparison. The three groups identified are underlined with the coloured line under the virus names (alphaormycovirus: black line, betaormycovirus: orange line, gammaormycovirus: green line).

We then searched for homologues of ElaOMV1 in our downy mildew libraries and grapevine powdery mildew libraries and assigned virus names to contigs (Table 1). Subsequently, we used the sequence of the RNA1-encoded putative proteins to search through tBLASTn in various assemblies from available fungal TSA from NCBI. This analysis allowed the identification of several new ormycovirus-like contigs coding for one putative protein each with at least partial conservation to ElaOMV1 protein sequence. Information about ORFan sequences and samples of origin are shown in Table 1. This group of viruses shares very limited identity in pairwise alignments, with the vast majority of them below 10 per cent; nevertheless, some subgroups sharing among themselves higher identity percentages are present and we provisionally named them alphaormycovirus, betaormycovirus, and gammaormycovirus (Fig. 3). As shown in Table 1, ormycovirus-like contigs were identified in a broad spectrum of fungi and oomycetes, including arbuscular mycorrhizal fungi, basidiomycetes, ascomycetes, and downy mildew-associated samples collected from grapevines. Interestingly, one additional ormycovirus-like contig was detected in the same isolate of powdery mildew infecting tomato where ElaOMV1 was initially found, but it was only clearly identified as ormycovirus after a focused reiterative search.

**Table 1.** List of ormycoviruses identified in the study. For each virus, NCBI ID or TSA ID is shown for each genomic fragment together with length information for each sequence.

Name	Acronym	genomic segment	length	ID
Erysiphe lesion associated ormycovirus 1	ElaOMV1	RNA1	3192	OM272927
		RNA2	1543	OM272928
Erysiphe lesion associated ormycovirus 2	ElaOMV2	RNA1	2478	OM272931
		RNA2	1875	OM272932
Erysiphe lesion associated ormycovirus 3	ElaOMV4	RNA1	2058	OM363731
		RNA2	1871	OM363732
Erysiphe lesion associated ormycovirus 4	ElaOMV4	RNA1	2548	OM272933
		RNA2	1881	OM272934
Plasmopara viticola lesion associated ormycovirus 1	PvlaOMV1	RNA1	3040	OM363727
		RNA2	1555	OM363728
Plasmopara viticola lesion associated ormycovirus 2^a^	PvlaOMV2	RNA1	2259	OM262448
Plasmopara viticola lesion associated ormycovirus 3	PvlaOMV3	RNA1	2406	OM363729
		RNA2	1771	OM363730
Plasmopara viticola lesion associated ormycovirus 4	PvlaOMV4	RNA1	2554	OM272935
		RNA2	1757	OM272936
Plasmopara viticola lesion associated ormycovirus 5	PvlaOMV5	RNA1	2406	OM272937
		RNA2	1788	OM272938
Plasmopara viticola lesion associated ormycovirus 6^a^	PvlaOMV6	RNA1	3325	OM262449
Plasmopara viticola lesion associated ormycovirus 7^a^	PvlaOMV7	RNA1	2956	OM262450
Starmerella bacillaris ormycovirus 1	SbOMV1	RNA1	2392	OM272929
		RNA2	1726	OM272930
Termitomyces ormycovirus 1	TOMV1	RNA1	2802	GFVP01007274.1
		RNA2	1976	GFVP01025626.1
Uromyces appendiculatus ormycovirus 2^a^	UaOMV2	RNA1	2661	GACI01004785.1
Ambispora leptoticha ormycovirus 1^a^	AlOMV1	RNA1	1266	GGIK01050282.1
Uromyces appendiculatus ormycovirus 1^a^	UaOMV1	RNA1	3034	GACI01002316.1
Auricularia auricula-judae ormycovirus 1^a^	AajOMV1	RNA1	3430	GFZV01012939.1
Erysiphe pisi ormycovirus 1	EpOMV1	RNA1	1844	GHEC01009306.1
		RNA2	1858	GHEC01007241.1
Ophiocordyceps sinensis ormycovirus 1	OsOMV1	RNA1	1564	GAGW01007749.1
		RNA2	1659	GAGW01007556.1
Puccinia striiformis ormycovirus 1^a^	PsOMV1	RNA1	1837	GAIR01011407.1

a Viruses where only RdRP-encoding genomic segments were identified.

Lastly, we used our results to search on two recently released large RNA viral databases (Edgar et al. 2022; Neri et al. 2022) where authors used an iterated homology approach to investigate a broad collection of metatranscriptomic samples and identified over 100k and 300k new viral RdRP, respectively. Interestingly, only the 300k virus database included several examples of ormycovirus RdRP-like that we included in our phylogenetic analysis (Supplementary Table S3) even though most have uncompleted coding sequences.

### 3.2 Detection of genomic fragments associated to ormycoviruses

The detection of an RNA2 associated with ElaOMV1 raised our interest in the identification of other fragments associated with the rest of the ormycovirus RNA1 described in Table 1. For this purpose, the putative protein from ElaOMV1 RNA2 was used as a query to search through BLASTX in the available databases. With this analysis, we have been able to identify only one contig from the libraries originated from *P. viticola-*infected grapevine leaves (Chiapello et al. 2020a), which can be associated to PvlaOMV1 since the abundance of reads present in different libraries corresponding to the two segments correlate quantitatively (Table 2) and qRT-PCR detected the presence of the two segments always in the same samples (Table 3). Given that the correlation between ElaOMV1 RNA1 and RNA2 is strongly supported by the conservation of their respective contig termini, our hypothesis is that homology between proteins encoded by RNA2 in the other members of the clades is not sufficient to make it detectable through the blast analysis. More investigation on the possible association between viral ORFans was made on the library from *S. bacillaris* isolate Cz12, (Crucitti et al. 2022) where we found SbOMV1. In the work mentioned above, *S. bacillaris* isolate Cz12, was also found infected with a mitovirus and a totivirus (called Starmerella bacillaris mitovirus 1, SbMV1 and Starmerella bacillaris totivirus 1, SbTV1) and another ORFan sequence called ORFan3; since, to our knowledge, no additional fragments were ever reported for fungal mitoviruses, we hypothesized an association between *S. bacillaris* ORFan3 (SbORFan3) and SbOMV1. To confirm this hypothesis, we needed to exclude the association of ORFan3 with the totivirus because satellite protein-coding segments can be associated with totiviruses (Bostian et al. 1980). For this purpose, we checked the complete collection of *S. bacillaris* isolates (Supplementary Table S2) for the presence of SbOMV1, SbORFan3, and the mitovirus and totivirus by specific qRT-PCR. Results showed that SbOMV1 and SbORFan3 were always found together infecting isolates Cz12, Cz16, and Cz25; SbMV1 was only detected in Cz12 isolate, whereas SbTV1 was detected in Cz12 and Cz25 isolates. Since Cz16 isolate only harbour SbOMV1 and SbORFan3, the association of these two fragments in the same viral genome seems to be supported (Table 4), confirming that SbOMV1 is indeed a bipartite mycovirus with SbORFan3 representing its RNA2 (Fig. 2B, Table 3). The RACE analysis confirmed conservation of the 5ʹ and 3ʹ termini between the two putative associated genomic segments (Supplementary Fig. S3).

The putative protein encoded by SbOMV1 RNA2 was then used to search for homologues through the tBLASTn analysis in the library where other ormycoviruses were found and interestingly, SbORFan3-like sequences were found in almost all the libraries investigated including PM-A library harbouring ElaOMV2 (Table 1). SbORFan3-like sequences were found in the downy mildew libraries, but mapping results for each Illumina library were not sufficient to resolve univocally the association between RdRP-encoding and SbORFan3-like contigs; thus, qRT-PCR primers were designed on ormycovirus contigs and on putative RNA2 to search for them in the single isolates from each of the downy mildew and powdery mildew pools (Table 3). Results allowed to confirm the strict association between the RNA1 of ormycoviruses and related RNA2 in all the viruses belonging to betaormycovirus and gammaormycovirus; the detection of RNA2 was performed on cDNA or total nucleic acid without reverse transcription for ElaOMV1 and 2 confirming results obtained for RNA1 segments (Fig. 1) and for SbOMV1 RNA2 (Crucitti et al. 2022). Taken together, these results support the evidence that ormycoviruses are bisegmented. We can hypothesize that when we could not associate the RdRP to an RNA2 is because of a more distant relation, which is not detectable through similarity searches thus preventing, for instance, the detection of RNA2 related to PvlaOMV2, 6, and 7 (Table 1 ).

**Table 2.** Reads Mapping to ormycovirus genomic fragments. Stranded mappings were performed to help the quantitative association between RNA1 and identified RNA2. For each NGS library, the total number of reads mapping on the genomic fragments is shown (column showing the library name) together with the number of reads mapping on the positive or negative sense of the sequence (column pos/neg).

Virus	length	DMGA	DMGApos	DMGAneg	DMGE	DMGEpos	DMGEneg	DMGD	DMGDpos	DMGDneg	PMA	PMApos	PMAneg	PMGA	PMGApos	PMGAneg
ElaOMV1 RNA1	3192	0	0	0	0	0	0	0	0	0	4810	3360	1450	0	0	0
ElaOMV1 RNA2	1543	0	0	0	0	0	0	0	0	0	2072	1814	258	0	0	0
ElaOMV2 RNA1	2478	0	0	0	0	0	0	0	0	0	3780	518	3262	0	0	0
ElaOMV2 RNA2	1875	0	0	0	0	0	0	0	0	0	2898	462	2436	0	0	0
PvlaOMV1 RNA1	3040	0	0	0	1492	1028	464	678	410	268	0	0	0	5216	3816	1400
PvlaOMV1 RNA2	1555	0	0	0	958	752	206	542	382	160	0	0	0	990	858	132
PvlaOMV3 RNA1	2406	2654	542	2112	244	58	186	3338	768	2570	840	184	656	3788	834	2954
PvlaOMV3 RNA2	1771	3160	702	2458	600	190	410	3002	888	2114	1060	210	850	2844	722	2122
PvlaOMV4 RNA1	2554	594	588	6	0	0	0	0	0	0	0	0	0	0	0	0
PvlaOMV4 RNA2	1757	1022	1020	2	0	0	0	0	0	0	0	0	0	0	0	0
PvlaOMV5 RNA1	2406	0	0	0	0	0	0	432	402	30	0	0	0	0	0	0
PvlaOMV5 RNA2	1788	0	0	0	0	0	0	314	276	38	0	0	0	0	0	0
ElaOMV3 RNA1	2058	0	0	0	14	0	14	0	0	0	0	0	0	4464	834	3630
ElaOMV3 RNA2	1871	0	0	0	64	12	52	18	2	16	0	0	0	2940	588	2352
ElaOMV4 RNA1	2548	0	0	0	0	0	0	0	0	0	0	0	0	1612	1568	44
ElaOMV4 RNA2	1881	0	0	0	0	0	0	0	0	0	0	0	0	4332	4244	88
	length	Yeast	Yeastpos	Yeastneg												
SbOMV1 RNA1	2392	132,412	13,262	119,150												
SbOMV1 RNA2	1726	164,614	14,410	150,204												

**Table 3.** Quantitative RT-PCR detection of ormycoviruses RNA1 and RNA2. Each ormycovirus was detected in single isolates from libraries where mapping data suggested its presence (Table 2). For each NGS library, the single isolates are shown on columns and Ct mean value for each genomic fragment is shown on rows. Sample metadata corresponding to powdery mildew libraries are available in Supplementary Table S1; metadata on downy mildew of grapevine libraries are published (Chiapello et al. 2020b) as supplementary material. Samples with a Ct higher than thirty eight were considered negative (blank in the table).

Library	Viruses	Isolates									
DMGA		17	18	20	25	30	73	82	86	91	119
	PvlaOMV4 RNA1							29,78			
	PvlaOMV4 RNA1							33,35			
	PvlaOMV3 RNA1			31,9			28,64	30,41	32,91	28,31	27,3
	PvlaOMV3 RNA2			32,76			29,82	30,19	32,29	28,4	27,19
		13	14	33	39	41	54	56	57	94	97
DMGE	PvlaOMV1 RNA1									26,52	30,3
	PvlaOMV1 RNA2									26,24	29,63
		5	11	35	36	38	40	45	96	110	113
DMGD	PvlaOMV5 RNA1				25,18						
	PvlaOMV5 RNA2				25,06						
		2	3	6	9	10	12	13	14		
PMGA	ElaOMV3 RNA1	32,5	21,23			20,03		23,11			
	ElaOMV3 RNA2	33,3	21,53			20,16		22,57			
	ElaOMV4 RNA1								24,87		
	ElaOMV4 RNA2								25,49		

**Table 4.** qRT-PCR detection of SbOMV1, SbMV1 and SbTV1. Isolates from the complete collection of *S. bacillaris* Cz are shown on columns and mean Ct value for each virus is shown on rows. The contig sequence previously called Starmerella bacillaris ORFan 3 is indicated in the table as SbOMV1 RNA2. Samples with a Ct value over thirty eight were considered negative (blank in the table).

Viruses	Cz1	Cz2	Cz3	Cz4	Cz6	Cz7	Cz12	Cz16	Cz21	Cz25	Cz26	CzGP
SbOMV1 RNA1							24,67	25,37		23,37		
SbOMV1 RNA2							23,95	24,41		23,49		
SbTV1	28,15		31,23		31,17		26,98			25,43		28,56
SbMV1	34,43						23,08					

Regarding the ormycoviruses identified in publicly available TSA from fungi, we could unequivocally identify the associated RNA only in some transcriptomes (Table 1), the exception being Puccinia TSA (GAIR01011407.1), where multiple fragments related to different ormycoviruses RdRP were found and the association with the putative RNA2 could not be established.

### 3.3 Structural prediction suggests that RNA1 of ormycovirus is an RdRP

Colab notebook was used to predict the structure of EaOMV1 virus and Saccharomyces 20S RNA narnavirus (ScNV-20S) using AlphaFold with default parameters. ScNV-20S was selected and used because it is the reference virus for narnaviruses, and a 3D structure determined although crystallography is available. In addition, narnavirus are a group of viruses with an RdRP size similar to ormycovirus. ScNV-20S RdRP protein was used as a template for building the model. The predicted structure for EaOMV1 showed a model confidence, in the RdRP palm domain region between 70 (confident) and 90 (very highly confident; data not shown).

**Figure 4.**
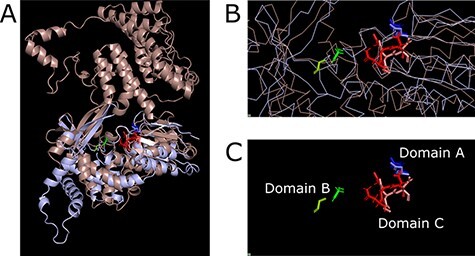
Three-dimensional alignment of ElaOMV1 (light-brown) and ScNV-20S (light-purple) RNA-dependent RNA polymerases. Panel A shows the alignment at a low magnification level, so it is possible to see the alignment of the overall protein structures (they are shown as cartoons). Panel B shows the alignment at a higher magnification level: it is possible to see the alignment of the palm domain (green, red, and blue belong to ElaOMV1; light-green, light-red, and light-blue belong to ScNV-20S; structures are shown as ribbons). In Panel C, the focus is only on conserved amino acids part of the catalytic triad in palm domains A, B, and C.

Although ElaOMV1 and ScNV-20S RdRPs showed low-sequence homology, the three-dimensional structures are highly conserved (Fig. 4A), especially nearby the amino acid residues that form the palm domain (Fig. 4B–C). Root-mean-square deviation of the atomic position of the two superimposed structures is 7.92. These protein regions, involved in catalysis and nucleotide selection, showed high identity proving that ElaOMV1 protein is, indeed, a putative RdRP. To confirm the result obtained, Swiss-model software was used to repeat the modelling analysis. In this case, the sequence from ElaOMV1 was used as a query on the web browser, obtaining as result a hit against the 2.4 angstrom resolution structure of the D346G mutant of the Sapporo Virus RdRP polymerase (Fullerton et al. 2007), thus confirming the conservation with a viral RdRP obtained with AlphaFold.

### 3.4 Further confirmation of the viral nature of ORFan contigs

**Figure 5.**
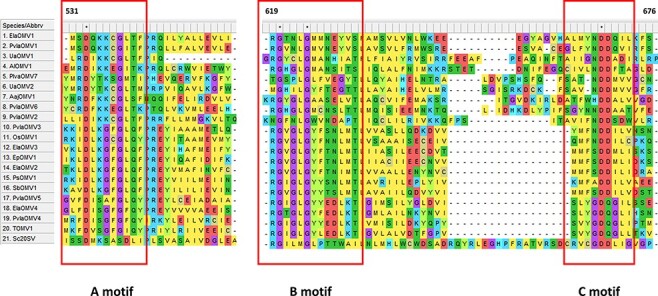
MAFFT alignment of the putative RdRP proteins encoded by ormycoviruses. Conserved residues from RdRP palm-domain motifs A, B, and C are highlighted in red boxes. Upper numbers show the position on the alignment of the first residue in the capture.

The absence of viral DNA in the ormycoviruses infected samples and the presence of reads mapping on both senses of the contigs (Table 2) was confirmed by analyzing the total nucleic acid from infected samples that were available in our laboratory (Crucitti et al. 2022), confirming a possible nature of ormycoviruses as RNA viral genome fragments. Based on the structural conservation with RdRP from 20S yeast narnavirus, we attempted a protein sequence alignment guided by the structural conservation to detect conserved regions within the RdRP palm domain, which represent the active sites of viral polymerases. MAFFT alignment (Fig. 5) allowed the identification of conserved regions among ormycoviruses, showing some motifs that could resemble the typical A-B-C motifs from RdRP Palm-domain that are essential for RNA replication activity (te Velthuis 2014). Even if not detectable through approaches based on similarity searches, partial conservation could be observed when aligning ormycovirus proteins to a known RdRP from a different viral taxon, this supporting an RdRP function for ormycovirus encoded proteins. In particular, the alignment displayed the conservation of the D residue in motif A, the two G residues in motif B and surprisingly in motif C, a wide array of catalytic triads was present, being NDD the most common, but also including the rare or unique ADD, SDD, HDD, and GDQ, this last catalytic triad being exclusive of gammaormycovirus (Fig. 5); uniqueness of the triad GDQ was confirmed analyzing the aligned 14,653 centroid RdRP1 sequences as defined in the serratus.io portal (Edgar et al. 2022).

### 3.5 Phylogenetic analysis

A reliable phylogenetic analysis that includes representatives of the five classes of RNA viruses could not be performed due to the very limited similarity of our group of viruses to those already included in the monophyletic tree. Nevertheless, Fig. 3 shows some conservation among the ormycoviruses we detected through similarity searches that allowed to perform the phylogenetic analysis. Ormycovirus-like sequences identified from the RVMT (Neri et al. 2022; Supplementary Table S3) were included in the analysis and named with the reference of the metatranscriptomic sample of origin.

**Figure 6.**
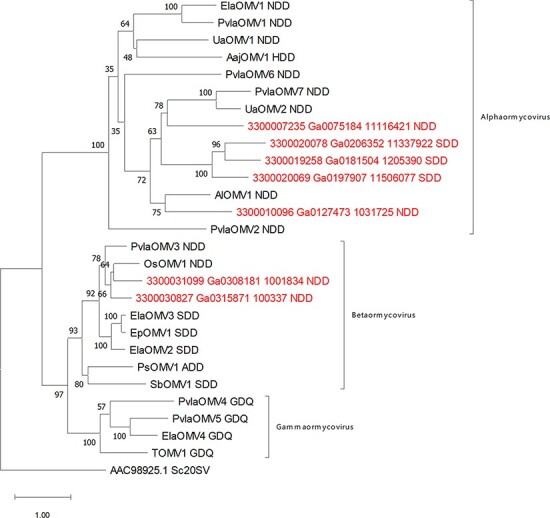
Maximum Likelihood phylogenetic tree of ormycoviruses. Putative RdRP from ormycoviruses was aligned using MAFFT and a phylogenetic tree was built through IqTree. *Saccharomyces cerevisiae* 20S narnavirus (ID: AAC98925.1) was used to root the tree. Sequences included are listed in Table 1 and Supplementary Table S3. Ormycoviruses identified from the RVMT database are shown in red with the reference ID of the metatranscriptome of origin. Next to each virus ID, the catalytic triad from motif C of the Palm-domain is displayed.

MAFFT alignments obtained from ormycovirus sequences were used to build a phylogenetic tree using the maximum likelihood method. Results (Fig. 6) showed that ormycovirus RdRP are divided in at least three different clades, with one clade (alphaormycoviruses, containing ElaOMV1) that seems more diverse compared to the other two. This result mirrors what has been suggested by the identity matrix scores (Fig. 3). In summary, our results support the evidence of the identification of at least three new groups of putative RNA viruses without a clear position in the RNA virus phylogeny for which we propose the name alphaormycovirus, betaormycovirus, and gammaormycovirus. To further confirm the RdRP nature of the putative proteins encoded by ormycoviruses belonging to different clades from the one hosting ElaOMV1, we submitted the SboMV1 putative RdRP for betaormycovirus and PvlaOMV5 for gammaormycoviruses on SWISS-model platform as described in ‘Detection of genomic fragments associated to ormycoviruses’ section. The automatic model search gave in both cases a viral RdRP domain as a result, confirming what was already observed for ElaOMV1. In addition, SbOMV1 also gave as result a helicase domain on the terminal portion of the putative protein while for PvlaOMV5, a DENN domain-containing protein and a PROXIMAL THREAD MATRIX PROTEIN were also selected as model templates on a different portion of the sequence, but these putative domains are not conserved among different viruses of the same group. Taken together, the data support the identification of at least three new viral groups with low homology to known RNA viruses. It is interesting to point out that all the viruses belonging to the betaormycovirus clade show the peculiarity of accumulating more reads mapping on the negative sense of both genomic fragments (Table 2), which is in contrast with mapping data observed for all the other ormycoviruses, where more reads mapping on the positive sense are observed; therefore, such property seems to be clade-specific.

Given that also RNA2 segments encode putative proteins that were found through homology searches, we also attempted protein alignment and the phylogenetic analysis. The identity matrix divided the putative protein into three groups that are equivalent to what is observed for the phylogenetic analysis performed on RdRP (Supplementary Fig. S4). No recognized conserved domains are present in these proteins and, therefore, their putative functions remain unresolved; nevertheless, some conserved motifs in the alignment of gamma and betaormycovirus-encoded ORF2 proteins are present (Supplementary Fig. S4).

### 3.6 Viral particle purification from ormycovirus infected yeast samples

Since SbOMV1 is the only ormycovirus available with a corresponding living biological sample in our collection, infected *S. bacillaris* isolates were used as a model to perform an initial biological characterization of the virus. Cz samples 12, 16, and 25 were used together with isolate Cz 21 (as negative control) to attempt the visualization of any viral particle under a transmission electron microscope. Raw yeast extracts were obtained by breaking the yeast pellet derived from 5 ml of liquid culture with a bead beater in 700 ml of 0.25 M phosphate buffer and isometric particles were observed only in the sample infected with the totivirus SbTV1 (Supplementary Fig. S5). Since this analysis only showed particles associated with SbTV1, we assumed that SbOMV1 could be a naked virus. We thus performed a purification protocol to detect SbOMV1 RNA distribution and to check for its enrichment in the soluble fraction of the yeast cellular components.

A partial cell fractionation protocol used before (Sato et al. 2020) to infer the nature of the viral RNA (naked, associated to proteins, encapsidated in virions) confirmed that true virions are not associated with this virus (Supplementary Fig. S6); nevertheless, most of the ormycovirus RNA precipitates in high-speed centrifugation pellets, suggesting that RNA-protein-association is likely.

## 4. Discussion

A recent paper that established a viral megataxonomy framework adopted by ICTV proposed a monophyletic tree for RNA and reverse transcribing viruses resulting taxonomically in a single realm called *Riboviria*. The classification is based on the conservation detected on the Palm-domain, which is found in all the viral RdRP and reverse transcriptase (Koonin et al. 2020). Nevertheless, recent findings showed several examples of newly discovered viruses whose phylogenetic position is unclear for the low conservation of the palm-domain (e. g. ambiviruses and orfanplasmoviruses) or for their specific genomic organization (like the splipalmiviruses) hosting the palm-domain on two different proteins (Sutela et al. 2020; Chiapello et al. 2020a; Chiba et al. 2021; Forgia et al. 2021; Linnakoski et al. 2021; Ruiz-Padilla et al. 2021) or the Quenyaviruses (Obbard et al. 2020). Specific bioinformatic approaches have allowed to include in recent versions of the tree also viruses with a permutated palm domain order, which have originated at different points in virus evolution (Neri et al. 2022). We believe that the viruses we here characterized are too distant from those present in currently available databases, and therefore a reliable phylogenetic tree cannot establish their position in the *Riboviria*. In facts, previous work has reached this conclusion for the Quenyaviruses clade (Obbard et al. 2020), even if the RdRP encoded by this clade is readily identified as such by the palmID tool implemented in serratus.io (Babaian and Edgar 2021; Edgar et al. 2022). By contrast, submission of putative ormycovirus RdRPs to the same portal fails to detect a palmID signature. It is also important to point out that, while ambiviruses were not detected by any homology approach, and their structural conservation with RdRP cannot be detected through alphafold (or equivalent protein structure search engines), the ormycoviruses we here characterized seem to be closer to known RNA viruses at the RdRP level because refined structural based searches indeed detected RdRP signatures. Ormycovirus-like RdRP were also found by the iterated RNA virus search pipeline recently developed (Neri et al. 2022) but were not assigned automatically to a new taxon. On the contrary, another recent work that characterized more than hundred thousand new RNA viruses did not include any ormycovirus-like sequence; direct comparison between the two databases has not been performed yet; therefore, we do not know if the discrepancy can be ascribed to a different search capability/sensitivity or to a larger metatranscriptomic dataset used. In this context, our independent characterization of ormycoviruses represents a necessary step in the direction of a broader exploration of the virosphere since even in the database that included ormycovirus-like sequences no host or taxonomy was associated to any of those mostly partial RdRP-encoding segments. Furthermore, although reads corresponding to ormycovirus sequences were present in public databases (NCBI) since 2020 (all the downy mildew of grapevine lesion metatranscriptomes), they were not detected in either of these two very large RNA virome characterization efforts.

Among the results described, it is worth to point out that in these rather diverse new clades grouped under the ormycovirus term we identified a variety of catalytic triads (amino acidic triplets) in the palm subdomain C. Together with the aspartic acid residue conserved in the A-motif, the same amino acid found in C-motif binds a metal cofactor and allows the synthesis of the new phosphodiester bond during RNA replication (Steitz 1998). The typical GDD (glycine, aspartic acid, and aspartic acid) triplet that is usually associated to viral RdRP, with some exceptions like the SDD triplet (circa 8 per cent of total) found in many negative sense, RNA viruses or the GDN triplet (circa 5 per cent of total), is not present in any of the ormycoviruses identified in this study, showing several alternatives like NDD, SDD, HDD, ADD, and GDQ. Some of these alternative triplets were previously unreported (te Velthuis 2014; Babaian and Edgar 2021; Edgar et al. 2022) and allow us to expand our knowledge on key components of the catalytic activity of viral RdRP.

The analysis of the orientation of the reads mapping on genome segments of SbOMV1 and other betaormycoviruses suggests a genomic replication strategy that is focused on higher accumulation of the negative sense of both RNA1 and RNA2 genomic strands. This feature is common to all the viruses belonging to the betaormycoviruses, but it is not shared by alpha- and gamma-ormycoviruses; furthermore, predominance of negative-strand accumulation occurred irrespective of the multiple libraries in which betaormycovirus were found. It would be tempting to attribute the betaormycoviruses to the negative sense RNA viruses (e.g. classify them as such), as indeed the higher accumulation of minus strand RNA is a typical feature of viruses with a negative-strand/ambisense genomic organization. Nevertheless, the definition of positive or negative sense RNA virus relies on the existence of viral particles (virions) and a negative-strand RNA virus encapsidates a negative sense genomic segment (antisense at least for the coding of the viral RdRP). In our case, neither true virions nor any structural signature of coat protein were found for the only virus that we could investigate biologically (SbOMV1), and therefore the definition can not be applied. Nevertheless, it is interesting to speculate that a constant higher-negative-strand accumulation during infection could be an intermediate step in the evolution of negative-strand RNA viruses. To our knowledge, such unbalanced negative strand higher accumulation was previously reported for viruses distantly related to totiviruses (Chiapello et al. 2020a) and to the splipalmiviruses previously characterized in fungi and oomycetes (Sutela et al. 2020). In some model viruses with positive-strand genomes, regulation of the ratio between positive- and negative-strands accumulations was thoroughly studied; such ratio seems to be important for switching from translation to replication modality occurring through preferential binding of virus or host factors to the plus or minus strand RNA (Dave et al. 2019). Although our data are inconclusive about the association of viral RNA to specific viral proteins, we cannot rule out that such association could be preferential to negative-strand RNA to form ribonuclear complexes similar to those typical of characterized negative-strand RNA viruses. Such ribonuclear complexes (in the absence of recognized coat proteins structural motifs) are, e.g. formed in the recently characterized polymycoviruses (Kanhayuwa et al. 2015), but in the case of other mycoviruses, naked RNA remains the most abundant RNA species as in the case of Hadaka viruses (Sato et al. 2020). The biological nature of fungal-like organisms and the most general horizontal mycovirus transmission modality (through hyphal fusion, without an extracellular step) allows mycovirus lifestyles relying on permanence and infectivity without the formation of true virions. This feature challenges some of the general concepts about viruses, such as those defining them based on the description of the nucleic acid that is encapsidated. Indeed, recent theories about virus origins suggest that viruses have formed as primordial replicators that recruited coat proteins in different events (Krupovic, Dolja, and Koonin 2019). In fungal-like hosts, the capsid is an accessory and often lost (or possibly never recruited). Besides, the newly found widespread occurrence of RNA viruses in prokaryotes (Neri et al. 2022) and the association between endo-bacteria and fungi, such as those occurring in arbuscular mycorrhiza (Bonfante and Desiro 2017), point to a possible route of horizontal transfer from bacteria to fungi that could be at the base of some virus diversity expansions such as those originating negative-strand RNA viruses. It is also noticeable that the recently described expansion of the virus diversity (Neri et al. 2022) altered the phylogenetic relationships that were inferred among the five monophyletic branches that comprise the classes *Lenarviricota, Pisuviricota, Kitrinoviricota*, *Duplornaviricota,* and *Negarnaviricota*, where the *Negarnaviricota* moved from a crown position in the previous analysis (Wolf et al. 2018, 2020), to a more basal position (next deepest compared to *Lenarviricota*) in the most recent and most comprehensive phylogenetic tree (Neri et al. 2022). The authors noticed the contrast of this basal position with the fact that negative-strand RNA viruses are absent from prokaryotes, archaea, and unicellular eukaryotes; considering that oomycetes and fungi are basal to the organisms that harbour the expansion of the *Negarnaviricota* (plants and animals) and that negative-strand-related mycoviruses were identified in both true fungi (Liu et al. 2014) and oomycetes (Botella et al. 2020; Chiapello et al. 2020a), we can here hypothesize that indeed abundant accumulation of negative-strand RNA during fungal infection can be at the origin of true, phylogenetically basal, negative-stranded RNA viruses.

Regarding the role of the RNA2-encoded ORFs present in the ormycoviruses, their relatively high identity conservation (identity percentages similar to those of the RdRP, compare Fig. 3 and Supplementary Fig. S3) in this very diverse group of viruses could point to a basal function accessorial to viral replication, but at the moment, the conserved motifs remain with an unassigned function.

The characterization of ormycoviruses demonstrates that our knowledge on viral diversity is still really limited, and that ad hoc studies of ORFans and viral dark matter remain a valuable tool to explore the virosphere. In this context, it is important to point out the value of the availability of SRA and TSA data that allowed us to confirm our findings with other transcriptomic samples independent from ours.

In conclusion, this study sets the basis of a broader characterization of viruses belonging to the three identified ormycovirus groups. Future studies would focus on the SbOMV1, infecting *S. bacillaris*, since it is the only virus entity, among the groups we characterized, present in an available biological sample where active replication is occurring and can be studied. In this regard, it will be interesting to study the biological effects of the virus on the yeast in mixed or single infection (as present in distinct isolates in our collection), particularly since this yeast is of oenological interest for some interesting properties, namely allowing the production of lower alcohol content and higher glycerol during fermentation (Englezos et al. 2017). This yeast species belongs to the Saccharomycetales and therefore can likely be developed into a genetically treatable host. Furthermore, given that budding yeast was shown to be a valuable host system (with a large available mutant collection) to study molecular details of virus–host interactions, also in case of viruses that do not infect fungi (Nagy 2008), we can envision exploiting also this system. To take advantage of these resources, we will perform studies with SbOMV1 through RNA transfection or through a reverse genetic approach based on cDNA infectious clones in *Saccharomyces cerevisiae*.

## Supplementary Material

veac038_Supp

## Data Availability

All data used for this work are available through accession numbers cited in the method section.
