# Supplementary material for: Three new clades of putative viral RNA-dependent RNA polymerases with rare or unique catalytic triads discovered in libraries of ORFans from powdery mildews and the yeast of oenological interest Starmerella bacillaris
Source: Virus Evol. 2022 Apr 23;8(1):veac038. doi: 10.1093/ve/veac038 (PMC9125799; doi:10.1093/ve/veac038)
Supplement: veac038_Supp [file veac038_supp.zip › SupplementaryMaterials_RevI.pdf]

Supp. Figure S1. Schematic representation of the bioinformatic pipeline used to detect and characterize viral ORFan

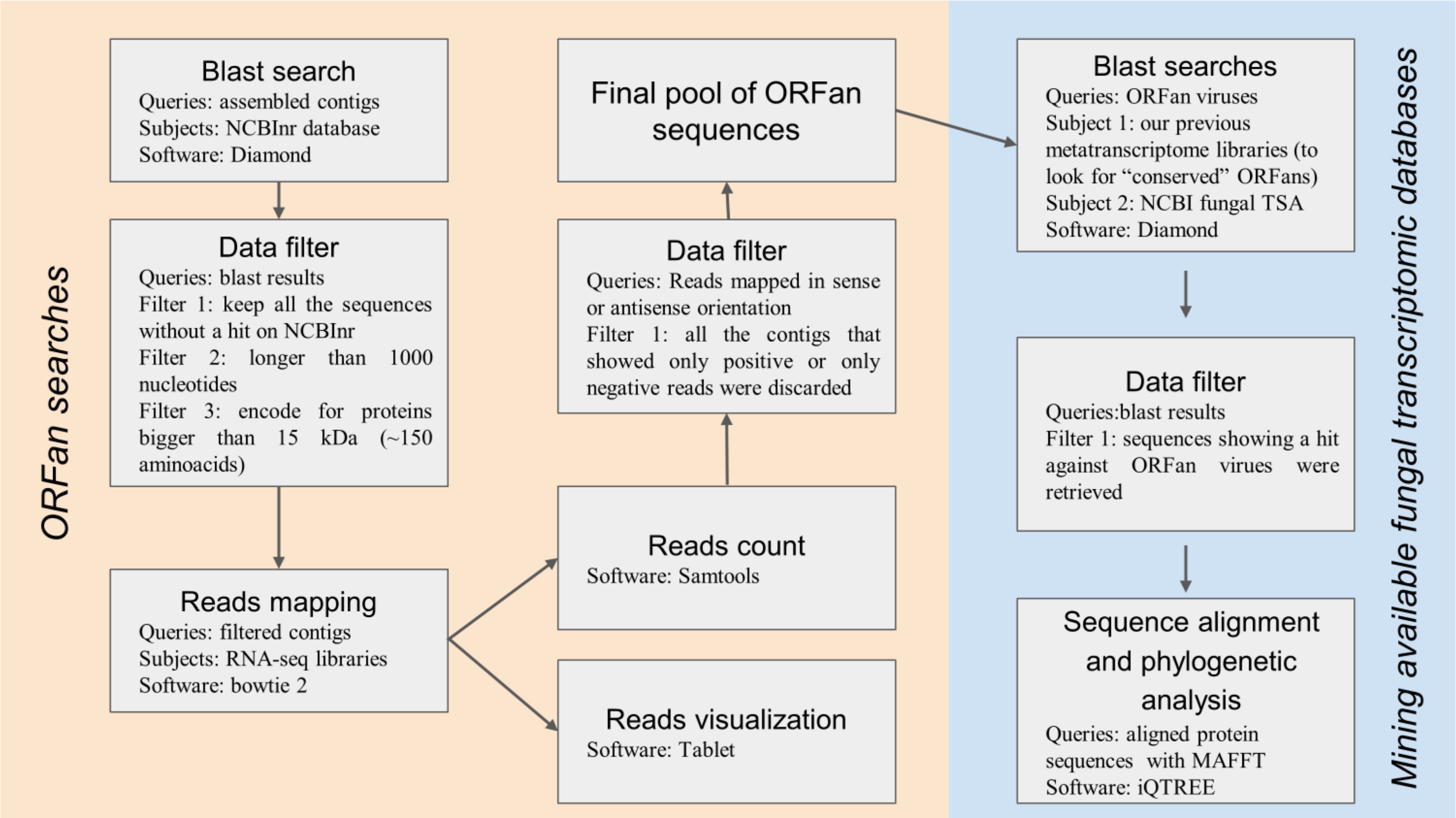

Supp. Figure S2. Electrophoresis gel of the PCR fragments obtained through amplification with primers complementary to 5' and 3' ends from ElaOMV1. The two bands are compatible with the expected sizes of the contigs obtained in silico for ElaOMV1 RNA1 and RNA2. Primer used for the amplification are listed in Supp. Table S4 (ElaOMV1_1_For, ElaOMV1_3192_Rev) and no template control is shown as NTC.

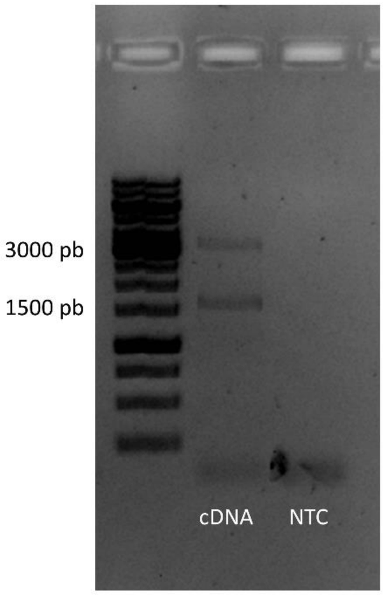

Supp. Figure S3. Ends conservation between RNA1 and RNA2 from ElaOMV1 and SbOMV1. Panel a shows a capture from the global alignment between ElaOMV1 genomic fragments. Panel b shows the same data from SbOMV1. Numbers represent the position on the genome. Only the 5’ and 3’ termini of the alignments are shown.

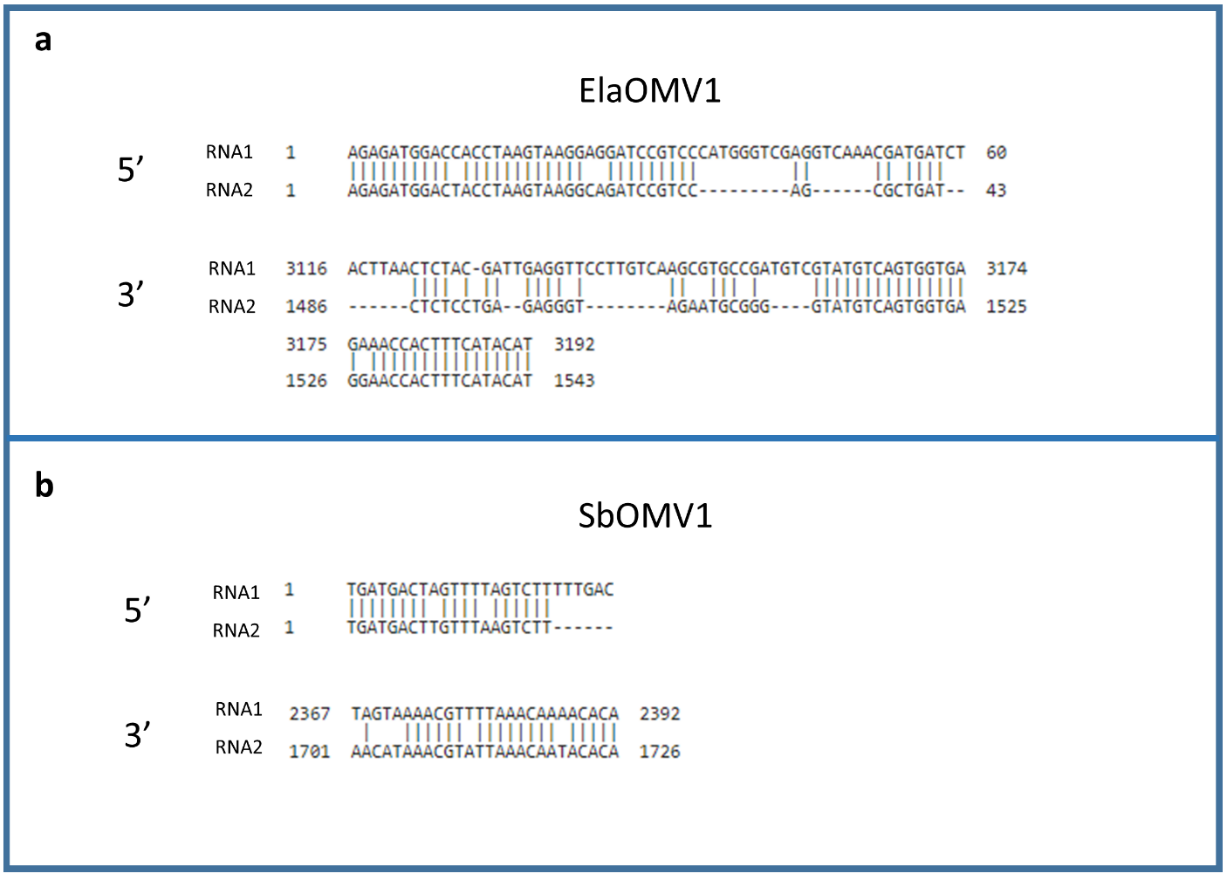

Supp. Figure S4. Ormycovirus RNA2 encoded protein comparison. Panel a shows region from MAFFT alignment of the RNA2 encoded putative proteins from betaormycoviruses and gammaormycoviruses. Putative proteins from alphaormycoviruses RNA2 were excluded from the analysis as no detectable homology was observed when comparing them to beta and gamma groups. The capture shows the region with higher residues conservation. Panel b shows pairwise identity matrix of putative proteins encoded by ormycoviruses RNA2. Putative proteins were aligned using MAFFT and pairwise identity comparison are shown in percentage. Names of the ormycoviruses are shown in different colors based on the groups identified through phylogenetic analysis.

a

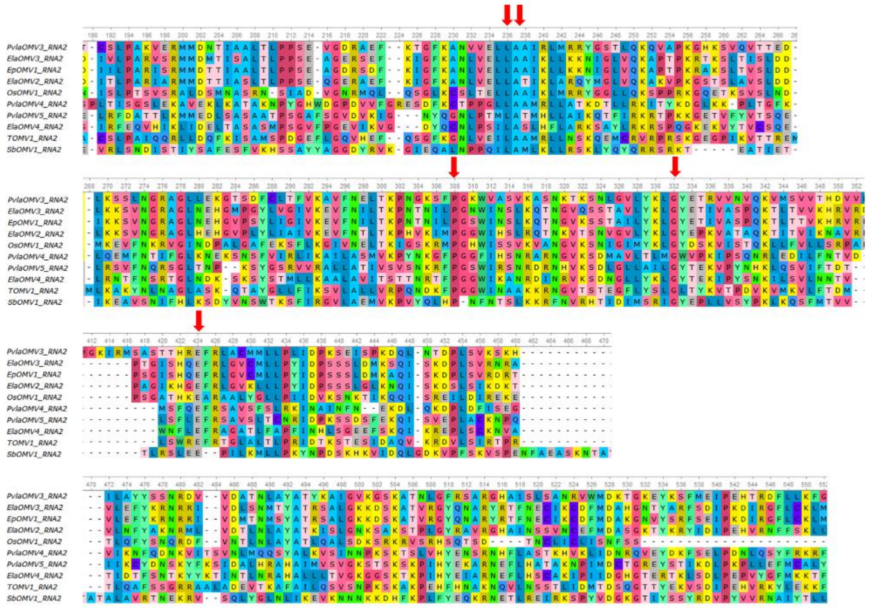

b

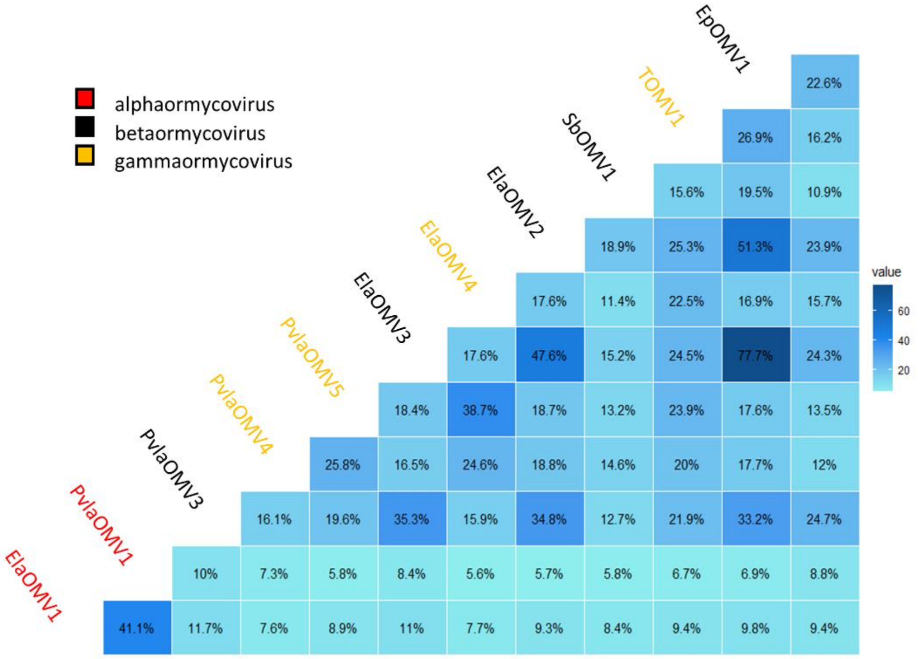

Supp. Figure S5. SbTV1 particles observed from isolate Cz25. Viral particles from the totivirus SbTV1 were observed through TEM on *S. bacillaris* isolate Cz25. The same isolate infected with SbOMV1 did not show any other conventional particle when performing raw purification protocol. White arrows indicate SbTV1 particles and size bars are shown lower left in each capture.

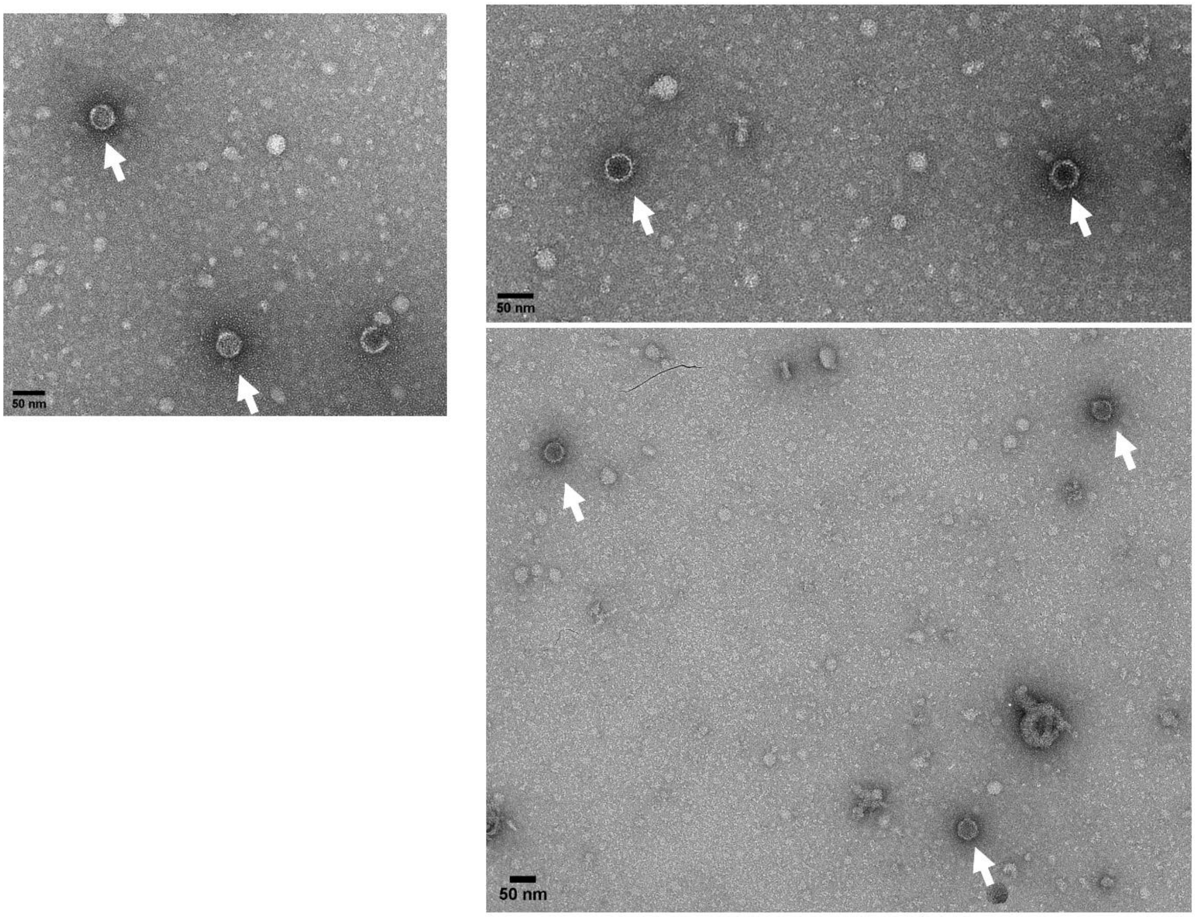

Supp. Figure S6. Relative distribution of SbOMV1 between different purification fractions. The figure shows RT-qPCR results for Cz12 and Cz25 isolates. Purification fractions are shown on the x axis with different colors indicated in the legend and grouped by target. Relative quantity is shown on y axis using sample A as control for each target. Panel a shows results obtained from Cz12. Panel b shows results for Cz25. Samples A are the raw lysates obtained from protoplasts. Sample P1 are the pellets obtained after raw lysate centrifugation at 20k g. Samples PU and SU are the pellets and supernatants obtained after ultracentrifugation at 100k rpm.

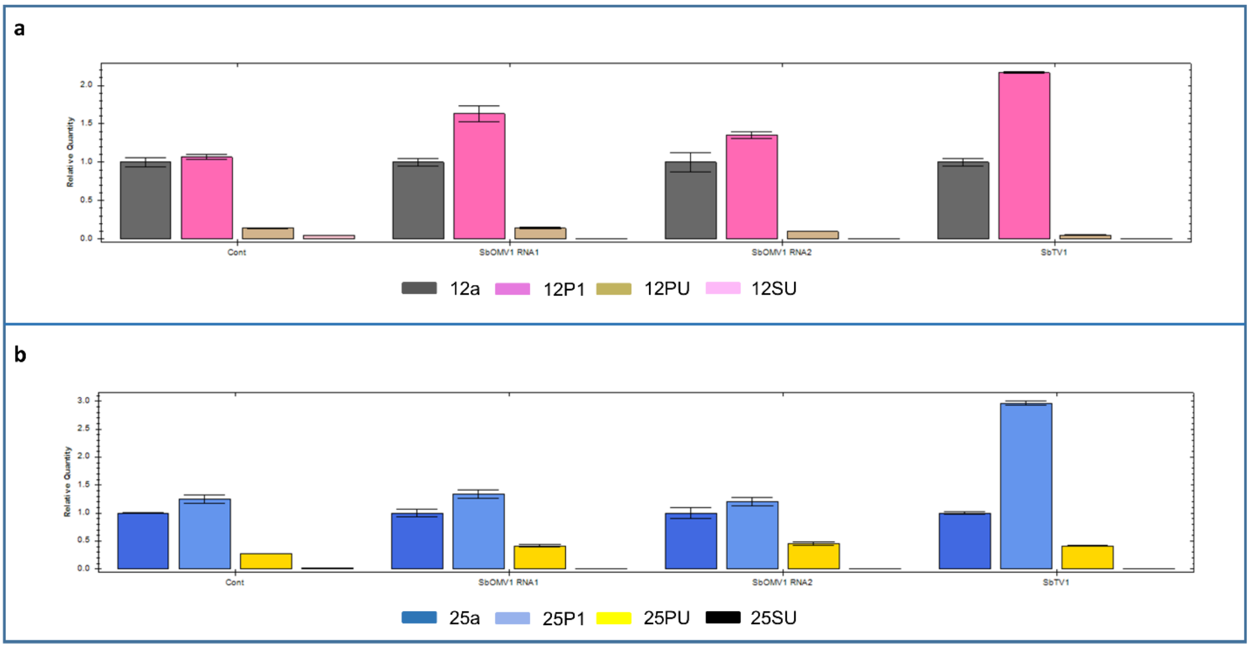

Supp. Table S1. List of single isolates contained in the libraries from powdery mildew of grapevine (PMGA) and vegetable crops (PM-A).

Library	Sample ID	Species/Variety	Host species	Sampling date	Region
PMG	2	Chardonnay	*Uncinula necator*	22/06/2018	Lombardia
PMG	3	Moscato	*Uncinula necator*	12/07/2018	Piemonte
PMG	6	ND	*Uncinula necator*	23/07/2018	Sicilia
PMG	9	Moscato	*Uncinula necator*	24/07/2018	Piemonte
PMG	10	Moscato	*Uncinula necator*	24/07/2018	Piemonte
PMG	12	Erbaluce	*Uncinula necator*	24/07/2018	Piemonte
PMG	13	Barbera	*Uncinula necator*	24/07/2018	Piemonte
PMG	14	ND	*Uncinula necator*	25/07/2018	Piemonte
PM-A	PMT1	Tomato	*Oidium neolycopersici*	12/07/2018	Piemonte
PM-A	PMT2	Tomato	*Leveillula taurica*	13/07/2018	Piemonte
PM-A	PMZ	Zucchini	*Podosphaera xanthii*	04/08/2018	Piemonte
PM-A	PMP	Pepper	*Leveillula taurica*	20/08/2018	Piemonte

Supplementary Table S2. List of *Starmerella bacillaris* strains from IRVO collection used for the study.

Strain	Source	Origin	Year of isolation	Brand
Cz1	Grape must cv. Catarratto	Marsala (TP)	2005	-
Cz2	Grape must cv. Catarratto	Marsala (TP)	2005	-
Cz3	Grape nust cv. Catarratto	Marsala (TP)	2005	BIOAGRO SRL
Cz4	Grape must cv. Catarratto	Marsala (TP)	2005	-
Cz6	Grape must cv. Catarratto	Marsala (TP)	2005	-
Cz7	Grape must cv. Catarratto	Marsala (TP)	2005	-
Cz12	Grape must cv. Catarratto	Marsala (TP)	2005	-
Cz16	Grape must cv. Catarratto	Marsala (TP)	2005	-
Cz21	Grape must cv. Catarratto	Marsala (TP)	2005	-
Cz25	Grape must cv. Catarratto	Alcamo (TP)	2005	-
Cz26	Grape must cv. Catarratto	Alcamo (TP)	2005	-
CzGP	Grape must (white berry)	Marsala (TP)	2013	-

Supp. Table S3. List of ormycovirus-like sequences found in RVMT database. Table shows RVMT ID (SeqID), IMGM ID and information on the metatrascriptomic source of the sample of origin from the JGI

SeqID	IMGM ID	Project
ND_408890	3300030827_Ga0315871_100337	Plant litter microbial communities from East Loma Ridge, Irvine, California
ND_141048	3300020078_Ga0206352_11337922	Corn, switchgrass and miscanthus rhizosphere microbial communities from Kellogg Biological Station, Michigan, USA
ND_230249	3300031099_Ga0308181_1001834	Bulk soil microbial communities from the East River watershed near Crested Butte, Colorado, United States
ND_136158	3300020069_Ga0197907_11506077	Corn, switchgrass and miscanthus rhizosphere microbial communities from Kellogg Biological Station, Michigan, USA
ND_128456	3300019258_Ga0181504_1205390	Peatland microbial communities from Minnesota, USA, analyzing carbon cycling and trace gas fluxes
ND_070387	3300007235_Ga0075184_11116421	Wastewater effluent complex algal communities from Wisconsin, to seasonally profile nutrient transformation and Carbon sequestration
ND_401447	3300010096_Ga0127473_1031725	Grasslands soil microbial communities from the Angelo Coastal Reserve, California, USA

Supp. Table S4. List of primers used in the study.

Primer Name	Sequence
LT_Suc_RealFor	GGGAGAGACAAGGTATGCGA
LT_SUC_RealRev	ATCTCTCGGCGCTTAATCGA
ElaOMV1_RNA1_For	TATCAGCCCCGGTGAACAAC
ElaOMV1_RNA1_Rev	GCGGTGATCAATGCCCAATC
ElaOMV1_RNA2_For	AGCCCTCCTCACAAAGGAGA
ElaOMV1_RNA2_Rev	GTCCTAGTCATTGACCCGCC
ElaOMV2_RNA1_For	GGAAACGCTTTTGCAGAGGG
ElaOMV2_RNA1_Rev	TGGATACGCACATTTCCCCC
ElaOMV2_RNA2_For	GGGTGTTTCCTTCACCCCTT
ElaOMV2_RNA2_Rev	TCTGACCCTCCGAGTCTGAA
PvlaOMV1_RNA1_For	GTTCTGTGGAGAACCCCGAG
PvlaOMV1_RNA1_Rev	GCGGCGGAATTTTTATCCCC
PvlaOMV1_RNA2_For	AGCGTGCGATTTCAAAAGGA
PvlaOMV1_RNA2_Rev	TAGTTTGTCGCCCCATCCTT
PvlaOMV3_RNA1_For	CGCAAGAACTGAGCGTGAAG
PvlaOMV3_RNA1_Rev	CTCGGTCACTGTTTCGTTGC
PvlaOMV3_RNA2_For	GAAGAAAAGAACGCCTCCCC
PvlaOMV3_RNA2_Rev	GCATTTACCCCGTTACGCAT
ElaOMV3_RNA1_For	GCATGTGGGCTACGTTAAGG
ElaOMV3_RNA1_Rev	ATTGCCCAGATTCGTCGTTG
ElaOMV3_RNA2_For	GCACCAACGCCTAAGAGAAC
ElaOMV3_RNA2_Rev	CCATGCTCGTTTAGTCCAGC
PvlaOMV4_RNA1_For	ACATAGGGTCTTCATGCCAGT
PvlaOMV4_RNA1_Rev	CACAGTCACCAAAAGGCACA
PvlaOMV4_RNA2_For	GGAGAGTAGCAGCTTGTCCT
PvlaOMV4_RNA2_Rev	AAGTTGTTTTGGAGGGGTCG
PvlaOMV5_RNA1_For	CAGGAGGGGTACTTCGATCC
PvlaOMV5_RNA1_Rev	TAGACGTGAACCCCTCCCTA
PvlaOMV5_RNA2_For	GTAGCGTGCAAGAACTCGTT
PvlaOMV5_RNA2_Rev	ATGGTGTCTGTCGGGAAGAG
ElaOMV4_RNA1_For	GGGCTCTTTTCCACTCAACG
ElaOMV4_RNA1_Rev	TGCAAAACATGTACACGCCT
ElaOMV4_RNA2_For	GCATCACCAATTCGAGTCGT
ElaOMV4_RNA2_Rev	CCTCCCGAAGATGTGATGGA
SbOMV1_RNA1_For	ACCGTCAATTCAGAGCGGTT
SbOMV1_RNA1_Rev	AACGGCCCTTTAAACCGAGT
SbOMV1_RNA2_For	TCGTCCGTGAGCTTGATCTG
SbOMV1_RNA2_Rev	ATCGTCAGCTCGGAACACTG
ElaOMV1_RNA1_358_rev	CATCTTTAAGCCTGGGAGGTTC
ElaOMV1_RNA1_240_Rev	CTCGTGACGCCACTTGACC
ElaOMV1_RNA1_2860_For	TGAATCGGTTGGACCTCCAA
ElaOMV1_RNA1_2925_For	CCAGGAACTGTTTCCGATGG
ElaOMV1_RNA2_320_Rev	CGCCTTCAGCAACTCTATTGC
ElaOMV1_RNA2_276_Rev	GGGTCATCTGCAAATGACTCT
ElaOMV1_RNA2_1226_For	AGCGCATAACGCGACTATG

ElaOMV1_RNA2_1295_For	GTCCTAGGCGAGAGTTTCTGA
ElaOMV1_RNA1_1_For	AGAGATGGACCACCTAAGTAAG
ElaOMV1_RNA1_3192_Rev	ATGTATGAAAGTGGTTTCTCACC
SbOMV1_RNA1_300_Rev	ATTCCTGAATCAAGATGGCATC
SbOMV1_RNA1_200_Rev	AAGGCTACCCACATGGATTC
SbOMV1_RNA1_2062_For	CGTACGCGAATCTACCAGTTA
SbOMV1_RNA1_2183_For	CGAAGAGCTGTACGACATGA
SbOMV1_RNA2_296_Rev	GTGATCTCAGATCAAGCTCACG
SbOMV1_RNA2_241_Rev	ATTAAATTCAACGCACGCGTC
SbOMV1_RNA2_1433_For	CGTGACGTTCCGTATGTAGT
SbOMV1_RNA2_1487_For	GGGAAGCCTAGAGATTTCTCCA
BlockedAdpt	Phosphate-GCATTCGACCCCGGGTT-AmC3
ComplAdapt	AACCCGGGGTCGAATGC
